# Structure and Mechanism of a Cold-Adapted Bacterial Lipase

**DOI:** 10.1021/acs.biochem.2c00087

**Published:** 2022-05-03

**Authors:** Florian van der Ent, Bjarte A. Lund, Linn Svalberg, Miha Purg, Ghislean Chukwu, Mikael Widersten, Geir V. Isaksen, Bjørn O. Brandsdal, Johan Åqvist

**Affiliations:** † Department of Cell & Molecular Biology, Uppsala University, Biomedical Center, SE-751 24 Uppsala, Sweden; ‡ Hylleraas Centre for Quantum Molecular Sciences, Department of Chemistry, University of Tromsø—The Arctic University of Norway, N9037 Tromsø, Norway; § Department of Chemistry—BMC, Uppsala University, Biomedical Center, SE-751 23 Uppsala, Sweden

## Abstract

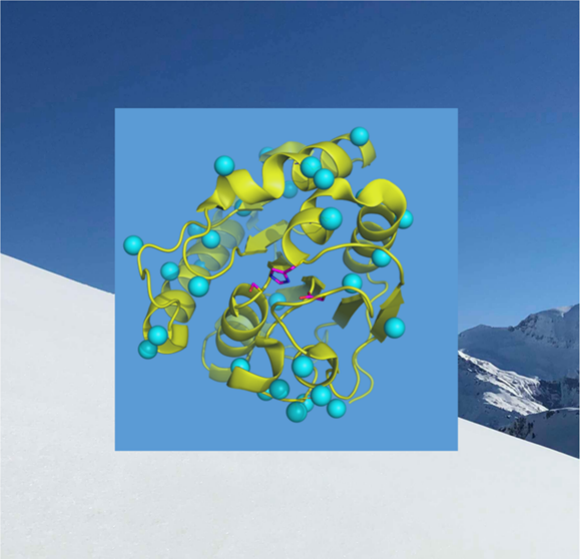

The structural origin of enzyme cold-adaptation has been the subject of considerable research efforts in recent years. Comparative studies of orthologous mesophilic–psychrophilic enzyme pairs found in nature are an obvious strategy for solving this problem, but they often suffer from relatively low sequence identity of the enzyme pairs. Small bacterial lipases adapted to distinctly different temperatures appear to provide an excellent model system for these types of studies, as they may show a very high degree of sequence conservation. Here, we report the first crystal structures of lipase A from the psychrophilic bacterium *Bacillus pumilus*, which confirm the high structural similarity to the mesophilic *Bacillus subtilis* enzyme, as indicated by their 81% sequence identity. We further employ extensive QM/MM calculations to delineate the catalytic reaction path and its energetics. The computational prediction of a rate-limiting deacylation step of the enzymatic ester hydrolysis reaction is verified by stopped-flow experiments, and steady-state kinetics confirms the psychrophilic nature of the *B. pumilus* enzyme. These results provide a useful benchmark for examining the structural basis of cold-adaptation and should now make it possible to disentangle the effects of the 34 mutations between the two enzymes on catalytic properties and thermal stability.

## Introduction

**Figure 1:**
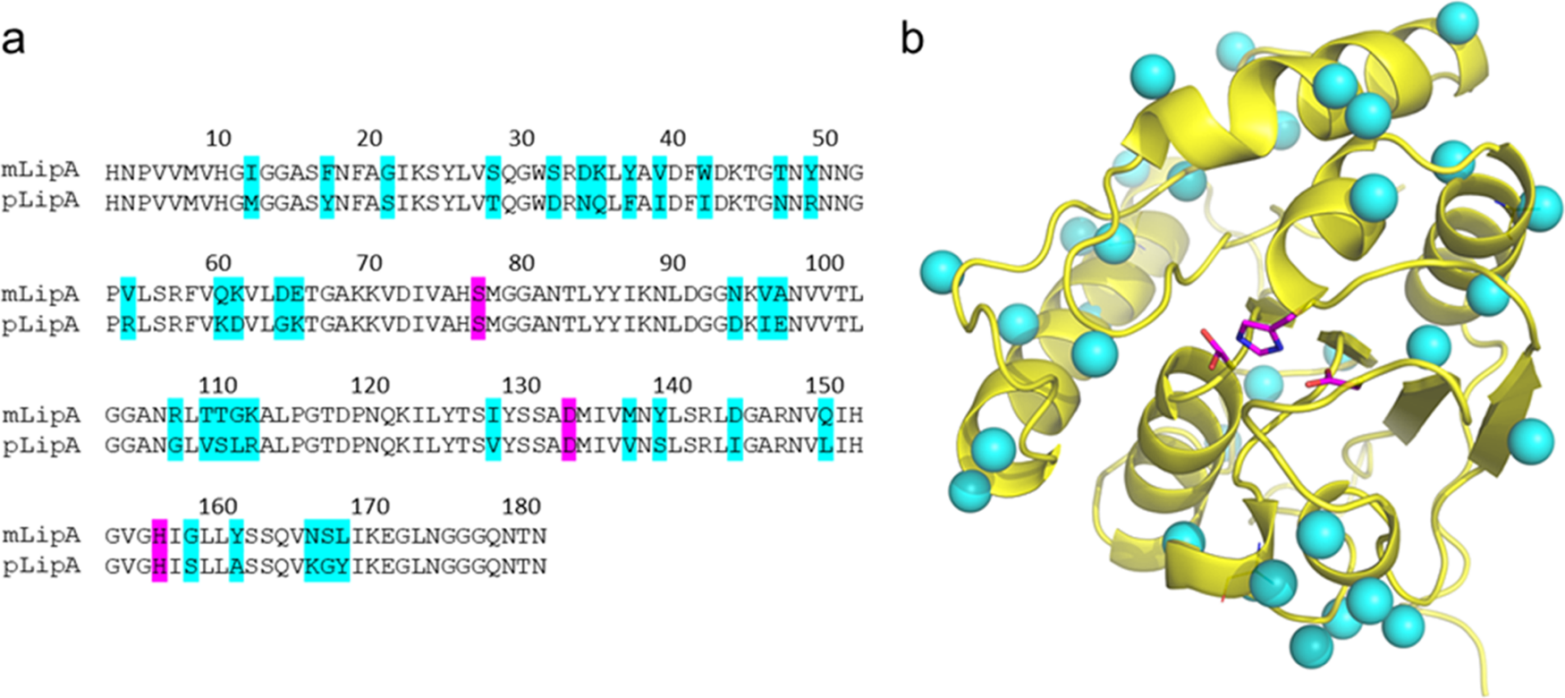
Amino acid sequences (a) and overall fold (b) of the mesophilic (mLipA) and psychrophilic (pLipA) lipases from *B. subtilis* and *B. pumilus*. Sequence positions that differ between the two enzymes are shown in cyan and the catalytic triad in purple. The three-dimensional (3D) ribbon diagram corresponds to the apo structure of pLipA determined herein.

Enzymes from psychrophilic species that live in permanently cold environments, such as polar regions and the deep sea, have been shaped by evolution to maintain high catalytic rates under these extreme conditions. Such cold-adapted enzymes are found in numerous species ranging from bacteria to invertebrates and polar and deep-sea fishes.^1−4^ They are of great interest both from a biochemical and evolutionary perspective and because they have considerable potential for biotechnological applications.^5^ What is particularly interesting with enzyme cold-adaptation is that sometimes relatively few amino acid mutations are needed to convert a mesophilic enzyme, with marginal activity at low temperature, to an efficient cold-active catalyst. A case in point here is the *Bacillus* lipases belonging to subfamily 4 of the largest bacterial lipase family (family I).^6^ Here, the small extracellular lipase A (LipA) from *Bacillus subtilis* is a well-characterized lipolytic enzyme that is used in the production of pure enantiomers in industrial applications due to its distinct substrate stereospecificity.^7,8^ A close relative of this mesophilic lipase (mLipA) is the corresponding psychrophilic BpL5 enzyme isolated from the arctic bacterium *B. pumilus* (denoted as pLipA herein).^9^ This enzyme has been reported to have a temperature optimum at 20–30 °C (about 10 °C lower than mLipA) and retain as much as 85% of its maximal activity even at 5 °C.^9^ At this temperature, mLipA is basically inactive.^10^ The thermal stability of pLipA has also been reported to be slightly lower compared to mLipA (about 5 °C).^9−11^ The sequence identity between pLipA and mLipA is about 81%, and only 34 out of the 181 residues thus need to be mutated to drastically alter the thermal properties. Interestingly, most of these mutations are located on the enzyme surface (Figure 1).

Attempts to engineer mLipA toward increased activity at low temperature via site saturation mutagenesis of loop regions have also been made.^10^ In that case, five point mutations with a significant activity increase at 5 °C were identified by the screening procedure. Two of the mutated residues (Phe17 and Met137) correspond to positions where mLipA and pLipA actually differ from each other, although the natural substitutions were not to glycine as in the laboratory screening. From that study, it is, however, noteworthy that the single M137G mutant, located in one of the surface loops covering the enzyme active site, was found to increase both *k*_cat_ and *k*_cat_/*K*_M_ by a factor of ∼3.5 (this residue is a valine in pLipA). Moreover, the combination of all five mutations showed some degree of additivity and increased this factor to about 7.^10^ The role of surface loops in enzyme cold-adaptation has also been highlighted in several computational studies of trypsin, elastase, triosephosphate isomerase, and α-amylase.^12−15^ In these cases, direct calculations of the temperature dependence of activation free energies demonstrated that a few surface loop point mutations can drastically change the Arrhenius plot for *k*_cat_ and the corresponding thermodynamic activation parameters (Δ*H*^⧧^ and Δ*S*^⧧^). The same phenomenon has also been observed experimentally.^16,17^ In the case of α-amylase, attempts to rationally redesign the psychrophilic enzyme from *Pseudoalteromonas haloplanktis* (AHA) toward mesophilic characteristics in terms of *k*_cat_ and *T*_m_ have also been reported.^18^ Also, in this case, primarily surface loop residues were chosen, based on the sequence comparison between AHA and the porcine pancreatic α-amylase (PPA). However, here, no single mutation was able to confer mesophilic characteristics to the psychrophilic enzyme, but the combination of seven mutations was found to increase *T*_m_ by about 8 °C and also bring *k*_cat_ closer to the value found for PPA.^18^

The bacterial lipases pLipA and mLipA clearly provide one of the best examples of psychrophilic–mesophilic enzyme pairs for exploring the structural end energetic basis of cold-adaptation by natural evolution. That is, these small globular and soluble enzymes (MW = 19.3 kDa) have a high degree of sequence identity but appear distinctly different with respect to temperature adaptation. Moreover, several crystal structures of mLipA in complex with various ligands have been determined.^8,19^ With the overall future goal of being able to both computationally and biochemically dissect the interplay between the ∼30 mutations responsible for cold-adaptation, we report here a combined experimental and computational characterization of the pLipA enzyme and its detailed reaction mechanism. High-resolution crystal structures of both the apo and holo forms of pLipA, the latter in complex with a covalently bound phosphoester derivative, have thus been determined and compared to the available mLipA structures. The mechanism and energetics of the acylation and deacylation pathway of the ester hydrolysis reaction are analyzed using extensive QM/MM calculations, and the prediction of a rate-limiting deacylation step is further verified by stopped-flow kinetic measurements. The temperature dependence of both the mLipA and pLipA reactions with a chromogenic substrate is also reported. With this data at hand, we now have an excellent benchmark for forthcoming in-depth analysis of the effects of different mutations on the cold-adaptation of pLipA. This will involve the construction of an efficient empirical valence bond (EVB) model of the catalytic reaction,^12−15^ which allows for much more extensive configurational sampling by molecular dynamics (MD) simulations and calculations of Arrhenius plots.^4^ Such a model can then be used for computational screening and prediction of the effects of single and multiple mutations, followed by experimental validation.

## Materials and Methods

### Protein Production

A synthetic *Bacillus pumilus* lipase gene construct corresponding to UniProt accession ID W8FKE7 was codon-optimized for expression in *Escherichia coli* and synthesized by GenScript. The mature peptide sequence, excluding the signal peptide, was subcloned into pET22b with cytosolic expression. The expression vector containing the pLipA gene was then transformed into Nico21(DE3) cells from New England Biolabs using standard heat shock protocols. Protein production was performed using ZYP-5052 autoinduction media, with a 5-h shaking at 37 °C before the temperature was reduced to 17 °C for overnight production. The protein was purified over three steps utilizing the encoded hexa-histidine C-terminal tag with an IMAC step, followed by desalting and then anion exchange with a strong ion exchanger. A salt gradient was used to elute the protein from the anion exchanger. For control experiments, the mesophilic *B. subtilis* lipase A was produced in the same manner.

### Crystallization, Data Collection, and Structure Determination

Paraoxon-ethyl oil was added to pLipA in the ratio of 1:100 before screening for crystallization conditions. Crystals for the pLipA-paraoxon complex were grown at room temperature from 0.1 M MES pH 6.5, 18–22% PEG 4000, and 0.1–0.5 M lithium citrate. The apo crystals were grown at 4 °C with the same conditions and vitrified in mother liquor mixed with 25% glycerol and 2.5 mM Orlistat inhibitor. The synchrotron measurements were carried out at the MX14.1 beamline at the BESSY II electron storage ring at the Helmholtz–Zentrum Berlin für Materialien und Energie, while the room temperature experiment was performed using a Bruker D8 Venture home source. Phases were obtained by molecular replacement using Phaser^20^ with a modified 1ISP^19^ structure as the search model. Refinement was done using phenix.refine.^21^

### Enzyme Activity Assay

Pre-steady-state experiments were performed with 80 μM 4-methylumbelliferyl butyrate mixed 1:1 with 4 μM pLipA, with 4000 samples collected over 0.2 s. A wavelength of 360 nm was used for excitation, and a filter of 400 nm to select for emission with an Applied Photophysics SX20 system. Steady-state kinetics was performed using a BioTek Synergy H1 plate reader kept at 25 °C. Briefly, 180 μL of *p*-nitrophenyl butyrate substrate dilutions from 5 mM to 39 μM was mixed with 20 μL of enzyme solution to a final enzyme concentration of 1.5 nM using the inbuilt liquid dispenser. The rates were monitored over 10 min to estimate the velocities. The velocities were fitted to the concentrations using nonlinear regression in GraphPad Prism v 6.0. Temperature-ramping experiments were performed using a Peltier-cooled qChanger6 (QNW) accessory for the Agilent Cary60 instrument. Here, 5 mM *p*-nitrophenyl butyrate dissolved in 50 mM potassium phosphate buffer pH 7.2 with 5% acetonitrile was mixed with the enzyme to a final concentration of 2 nM, and the temperature was ramped with a gradient of 1 °C/min. The absorbance was followed at 405 nm.

### Computational System Preparation

The new crystal structure was prepared for simulation using Schrödinger Maestro,^22^ protonation states were determined using PROPKA,^23^ and the hydrogen bonds were optimized. Two systems were prepared, the enzyme-substrate complex with *p*-nitrophenol butyrate bound in the binding site and the acyl-enzyme with the ester of Ser78 and butyric acid. Using QwikMD,^24^ both systems were prepared, the protein was solvated in a box with a 12 Å buffer to the edges and neutralized and brought to a 0.15 M salt concentration using sodium and chloride ions. All MD simulations were run using the NAMD molecular dynamics package,^25^ the CHARMM36 protein force field^26^ and the TIP3P water model.^27^ Parameters for the 4-nitrophenol butyrate and ester side chain were generated using cGenFF.^28^ A direct 12.0 Å cutoff was used for nonbonded interactions with long-range interactions described by the particle mesh Ewald method.^29^ A switching function for Lennard–Jones interactions was applied between 10.0 and 12.0 Å. All MD simulations were performed using a 2 fs time step in the NPT ensemble at 1 atm, using a Langevin dynamics thermostat (damping coefficient 1 ps^–1^) and a Nosé–Hoover Langevin piston barostat.

The simulation protocol first involved 2000 steps of energy minimization, after which the system was heated from 60 to 300 K over the course of 288 ps MD simulation by incrementing the temperature by 1 K every 600 time steps (1200 fs). The system was then equilibrated at 300 K for 1 ns before production MD simulation. The heating of the system and equilibration were performed using 1.0 kcal mol^–1^ Å^–2^ harmonic positional restraints on all protein backbone atoms. For both the enzyme-substrate complex and acyl-enzyme, five replicate MD simulations were run, each comprising a 10 ns trajectory, yielding a total of 50 ns production MD per system (Figure S1).

From the resulting trajectories, snapshots were selected to be used as starting points for the QM/MM simulations. Cutoffs were used to remove nonreactive snapshots and in the acyl-enzyme simulations, and MDtraj^30^ was used to find frames with a water molecule in position for the nucleophilic attack on the ester carbon. The cutoff criteria for acylation were: Ser77Oγ–substrate C1 ≤ 3.0 Å, Ser77Oγ–His156Nε2 ≤ 3.0 Å, and His156Nδ1–Asp133Oδ2 ≤ 3.0 Å. The corresponding criteria for deacylation were WatO–substrate C1 ≤ 2.9 Å, WatO–His156Nε2 ≤ 2.9 Å, and His156Nδ1–Asp133Oδ2 ≤ 3.0 Å. From the MD frames satisfying the reactive geometric criteria, 20 snapshots were selected at random to be used in the QM/MM calculations. To prepare the systems for QM/MM calculations, the Ser77 hydroxyl oxygen was taken as the origin and all waters beyond 25 Å were removed to get a sphere centered on the QM region. The ORCA program (version 5.0.1) was used for all QM/MM calculations.^31^

### QM/MM Calculations

The systems were partitioned into three regions, QM, active MM, and inactive MM. The QM region consisted of the side chains of the three residues making up the catalytic triad (Ser77, His156, and Asp133) and the substrates. As one of the backbone amides that makes up the oxyanion hole corresponds to Met78 and is adjacent to Ser77, we extended the QM region of Ser77 to include this amide and the atoms connecting it to the serine side chain. For the acylation reaction, the *p*-nitrophenyl butyrate substrate was included in the QM region, and for the deacylation reaction, the Ser77-acyl group and a single water molecule in position for nucleophilic attack were included. Different sizes for the active region were used for different steps of the protocol. First, a minimization with a large active region of 10 Å around the QM region was performed with HF-3c/MM,^32^ constraining the QM system to an approximate tetrahedral intermediate. Then, the active region was set to include all residues within 2.8 Å of the QM region, and the QM region was constrained toward the expected minima and maxima using PBEh-3c/MM as the energy function.^33^ For the minima, a second unrestrained tight optimization was performed until the structures converged. These minima were used as starting and end points for initial nudged elastic band (NEB) paths.^34^ The approximate transition states (TSs) were used as intermediate structures for the generation of the initial paths. An initial climbing image optimization of the NEBs was performed using the FIRE optimization algorithm, followed by a tight-NEB-TS optimization using the LBFGS algorithm. In some cases, this second optimization was not successful and then the zoom-NEB-TS method was used to optimize these replicates.^31^ When stationary points were successfully converged for 20 replicates for each system, thermal corrections were obtained from frequency calculations on the QM region plus atoms in the MM region with a covalent bond to the QM region. The chain-of-spheres (RIJCOSX) method^35^ was used to speed up hybrid density functional theory (DFT) calculations, and the resolution of identity (RI) approximation^36^ was used to speed up DSD-PBEB95 calculations, with def2-QZVPP/C an auxiliary basis set.^37^

## Results and Discussion

### Crystal Structures of pLipA

The apo structure of pLipA had reflections up to a resolution of 0.87 Å with *R*-factors (*R*/*R*_free_) of 11.7/13.5% after refinement (Table 1), which is among the highest resolution models available for lipases/esterases. A structure with a phosphorylated derivate of the catalytic Ser78 residue was also obtained by treatment with paraoxon. Here, the highest resolution data was collected up to 1.5 Å at room temperature, with *R*-factors of 12.6/16.0% after refinement. A structural comparison of the 21 *B. subtilis* LipA structures in the Protein Data Bank (identified by their UniProt accession number P37957) shows a highly conserved mainchain structure with an average root-mean-square deviation (RMSD) for the Cα-atoms of 0.3 Å aligned to the 1.3 Å resolution structure with PDB code 1ISP.^19^ The atomic resolution apo structure of the *B. pumilus* LipA also shows a high degree of conservation of its mainchain structure, with an RMSD value of 0.4 Å to 1ISP. The differences between these two structures are mainly located on the enzyme surface (Figure S2). The holo structure with the phosphorylated serine derivate gives an almost identical RMSD value of 0.4 Å when compared to 1ISP. Overall, the difference in structures between mLipA and pLipA are minimal when taking the 81% identity into account. Further, the phosphoester derivate gives critical insight into the expected transition state for the deacylation step in the hydrolysis of esters, as the phosphate has a tetrahedral geometry. Because of its resemblance to the expected transition state, this structure was used as a starting point for the QM/MM calculations.

**Table 1:** Data Collection and Refinement Statistics^a^

	**pLipA phosphate ester**	**pLipA apo**
PDB entry	7R1K	7R25
wavelength	1.5418	0.9184
resolution range	20.9–1.50 (1.55–1.50)	35.1–0.87 (0.90–0.87)
space group	*C*2	*P*2_1_
unit cell (Å/°)	*a =* 57.4, *b* = 42.8, *c* = 62.7	*a* = 35.1, *b* = 54.7, *c* = 41.0
	α = 90, β = 91.3, γ = 90	α = 90, β = 92.7, γ = 90
total reflections	296 666 (18 263)	634 145 (5885)
unique reflections	47 178 (4727)	103 387 (1919)
redundancy	6.3 (3.9)	6.1 (3.1)
completeness (%)	98.7 (98.5)	81.8 (15.2)
*I*/σ(*I*)	9.4 (1.1)	30.5 (2.2)
*R*_merge_	0.123 (1.492)	0.028 (0.445)
CC_1/2_	0.997 (0.399)	1 (0.786)
CC*	0.999 (0.755)	1 (0.938)
reflections used in refinement	47 149 (4710)	103 381 (1919)
reflections used for *R*_free_	2292 (231)	2100 (39)
*R*_work_	0.143 (0.298)	0.1117 (0.239)
*R*_free_	0.166 (0.310)	0.1335 (0.292)
no. of non-hydrogen atoms	1516	1743
macromolecules	1391	1447
ligands	40	33
solvent	99	273
RMSD		
bond lengths (Å)	0.009	0.009
bond angles (°)	0.90	1.05
ramachandran (%)		
favored	97.77	97.80
allowed	2.23	2.20
outliers	0.00	0.00
rotamer outliers (%)	0.00	1.29
clashscore	2.12	5.07
average *B*-factor	17.56	12.66
macromolecules	16.73	10.76
ligands	13.71	25.99
solvent	30.23	21.61
number of TLS groups	11	

a Statistics for the highest resolution shell are shown in parentheses.

**Figure 2:**
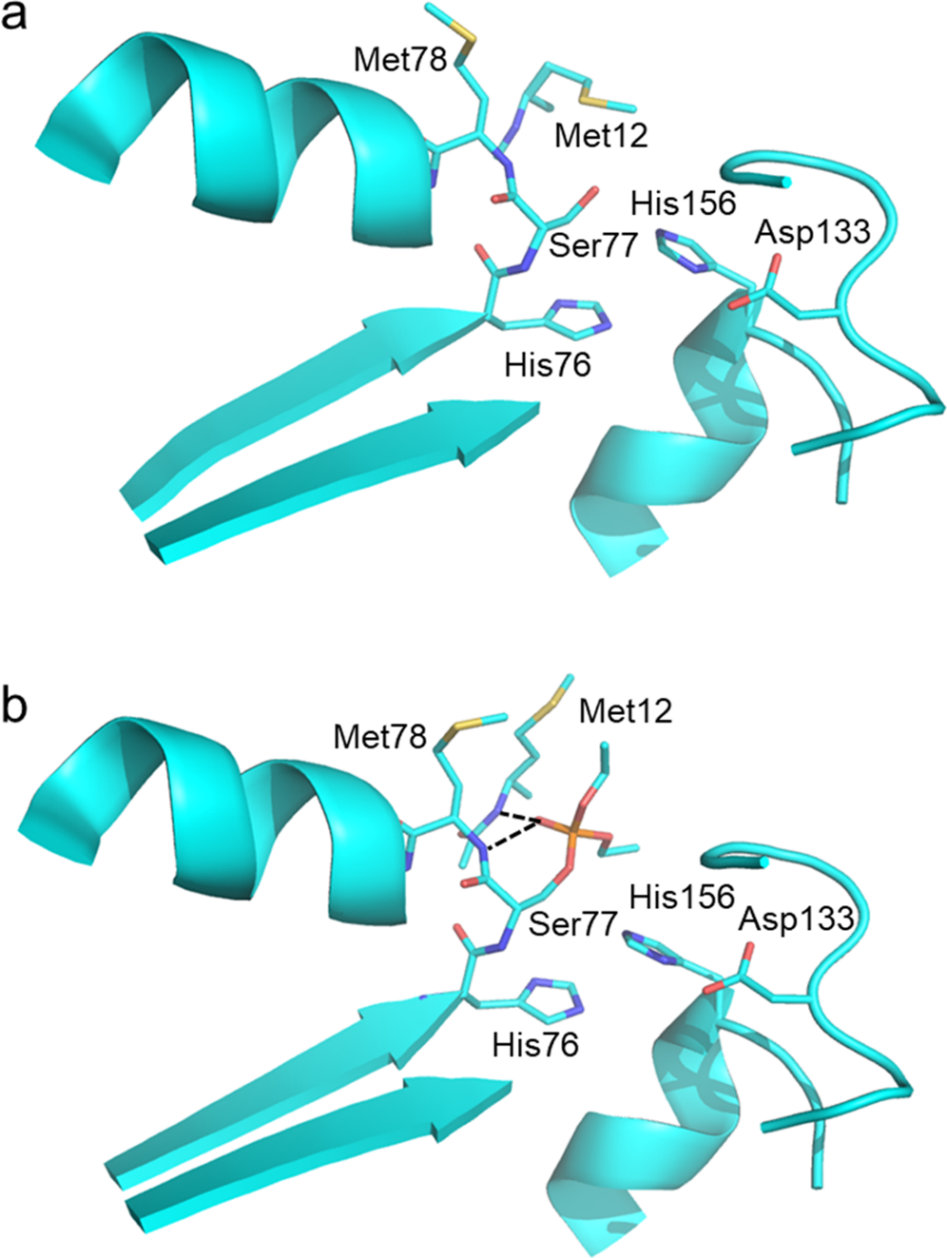
Comparison of the active site in the (a) apo and (b) holo forms of pLipA. The reaction of pLipA with paraoxon-ethyl ester yields a stable covalent intermediate as a suicide inhibitor. Met12 and Met78 show the largest conformational changes upon inhibitor binding.

A structural comparison of the two structures of pLipA using pdbeFold^38^ also gives an RMSD of 0.4 Å over 176 residues. The largest structural variations were observed for Asn34, Lys44, Lys65, Leu111, Asn120, Met134, and Ile157 based on the PDBeFold analysis. These residues are all located at the enzyme surface and would likely be sensitive to the different crystal packing arrangements between the two forms and the overall flexibility. That is, differences due to flexibility are likely to be magnified here since the phosphorylated serine form was collected at room temperature, while the high-resolution apo structure was collected at 100 K with synchrotron radiation. Although the apo structure was also soaked with the lipase inhibitor Orlistat, no density was observed for this compound. The binding of the paraoxon ester leads to a conformational change of the side chains of Met12 and Met78, which would otherwise have steric clashes with the substrate in their apo-conformation. It is the mainchain amides of these two residues that form the oxyanion hole (Figure 2), which facilitates catalysis, and in mLipA Met12 corresponds to an isoleucine while Met78 is conserved. This I12M mutation is, in fact, the only substitution in the vicinity of the substrate binding site.

### QM/MM Calculations of the Reaction Pathway

**Figure 3:**
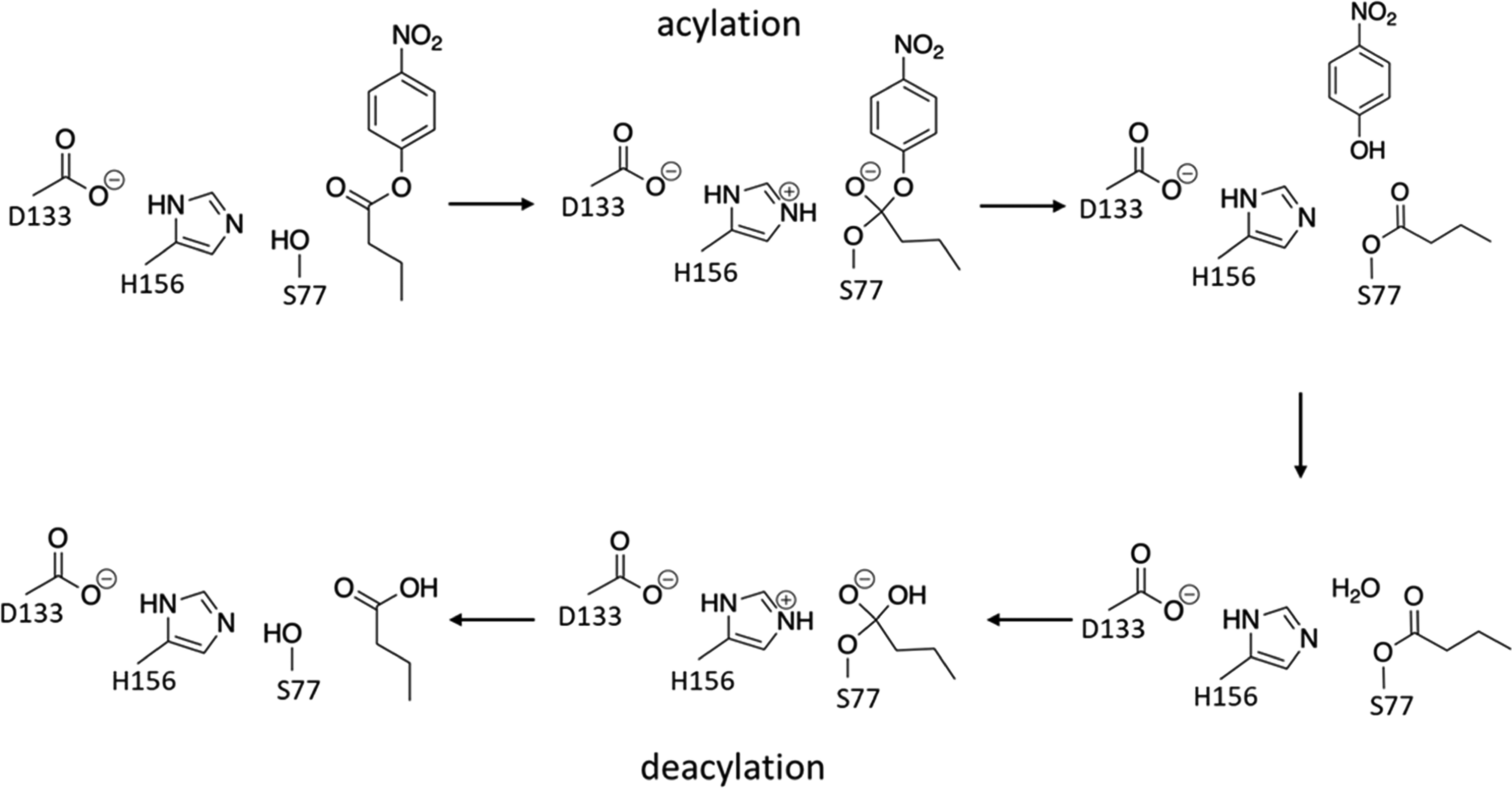
Overall reaction mechanism of the two lipases consisting of an acylation step that yields a covalent enzyme-substrate intermediate, followed by a deacylation step where this intermediate is hydrolyzed by a water molecule. Ser77 is the nucleophile in the initial attack on the substrate, and His156 acts as a general base in the nucleophilic displacements of both steps. Note that the acylation step is found to be concerted herein.

**Figure 4:**
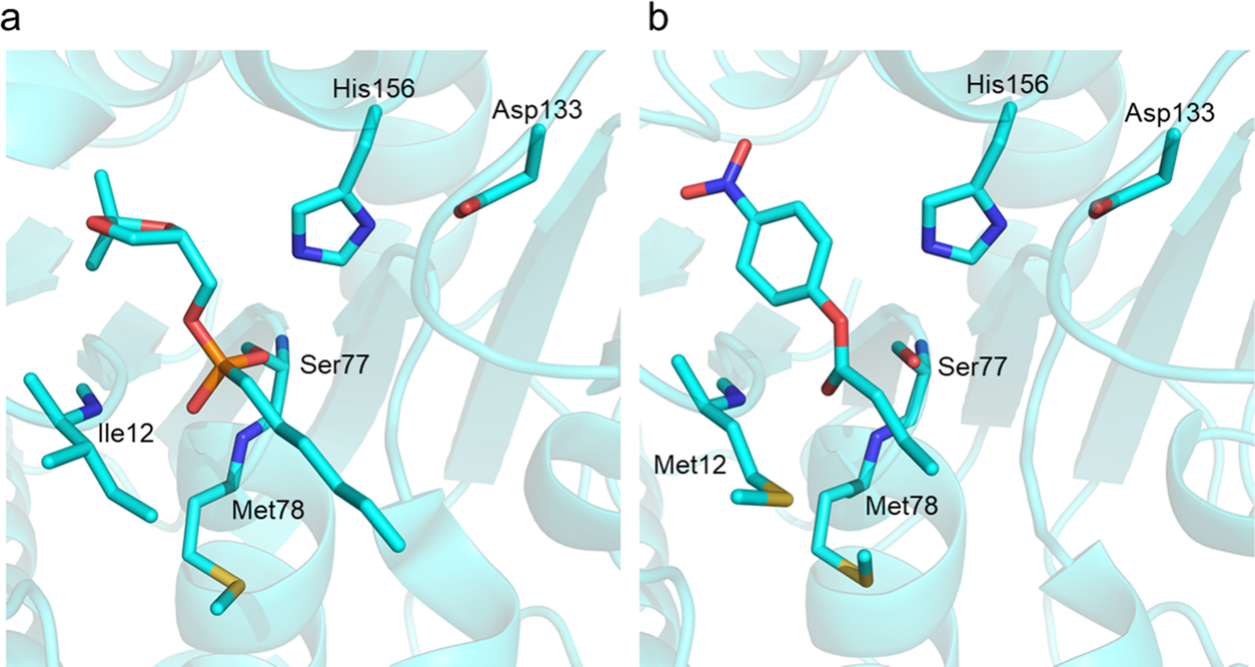
(a) View of the crystallographic structure of the complex of mLipA with a covalent 1,2-*O*-isopylidene-*sn*-glycerol phosphonate inhibitor bound to Ser77 (PDB code 1R4Z).^8^ (b) Initial model of the substrate *p*-nitrophenyl butyrate in the pLipA active site used in the MD simulations.

Our first objective was to determine the detailed mechanism of action of the cold-adapted lipase by means of QM/MM calculations. It may be noted here, of course, that the reaction mechanisms of pLipA and mLipA are not expected to be different. However, there appear to be no QM/MM calculations on this lipase family published before and it is thus essential to clarify the detailed mechanism and energetics with the commonly used *p*-nitrophenyl butyrate (PNP-C4) substrate to eventually construct an efficient EVB model.^4^ The reaction can be split into two steps typically found in esterases and proteases, showed in Figure 3, and these two steps were modeled separately. We started by running five independent 10 ns MD simulations of the fully solvated enzyme-substrate complex in a periodic box, with PNP-C4 bound in the active site. This substrate has been shown earlier to be efficiently cleaved by the pLipA enzyme.^9^ The initial structure in these simulations was based on our phosphorylated complex, where the positioning of the *p*-nitrophenyl group was guided by an earlier published mLipA structure with a covalent 1,2-*O*-isopylidene-*sn*-glycerol phosphonate inhibitor, as shown in Figure 4.^8^ In these simulations, a flat-bottom distance restraint (*d >* 3.5 Å, *k* = 10 kcal/mol Å^–2^) was applied between the attacking Ser77 hydroxyl oxygen and the ester carbon atom of the substrate to ensure good sampling of the reactive noncovalent complex since substrate binding is rather weak as judged from the experimental *K*_M_ value.^9^ These trajectories were then used to select snapshots for QM/MM calculations. A number of inactive substrate conformations were found in the MD trajectories, where either the ester carbonyl oxygen was not correctly positioned in the oxyanion hole made by Met12 and Met78 or Asp133 was turned away from His156. Such inactive frames were discarded using distance cutoffs.

For the deacylation reaction, unrestrained MD simulations were used as there is no possibility for substrate dissociation and the acylated Ser77 also had a stable conformation. Frames where a water molecule was in position for nucleophilic attack on the acyl-ester and His156 was in position for deprotonating the water molecule were selected using distance cutoffs. From these frames, a random subset of 20 was selected to be used for the QM/MM calculations. PBEh-3c was used as a reliable and affordable hybrid functional to perform initial structure minimizations.^33^ After initial scans, we could not find a stable tetrahedral intermediate in the acylation reaction and thus decided to use a single nudged elastic band (NEB) to find the minimum energy path between the Michaelis complex and acyl-enzyme. For the deacylation step, a stable tetrahedral intermediate was identified and optimized and two NEBs were used to identify the transition states. Based on published benchmarks^39^ the RI-DSD-PBEB95-D3BJ double hybrid functional^40^ with the def2-QZVPP basis set^41^ was selected as a method with a high enough accuracy to calculate final barriers. It was also used as a reference to select a hybrid method with a medium-sized basis set to be used for final geometry optimizations and frequency calculations (Figure S3). For all transition states, a single imaginary frequency was found with wavenumbers 515.5, 590.7, and 573.7 cm^–1^ for TS1, TS2, and TS3, respectively, and standard errors <36 cm^–1^ among the replicas.

**Figure 5:**
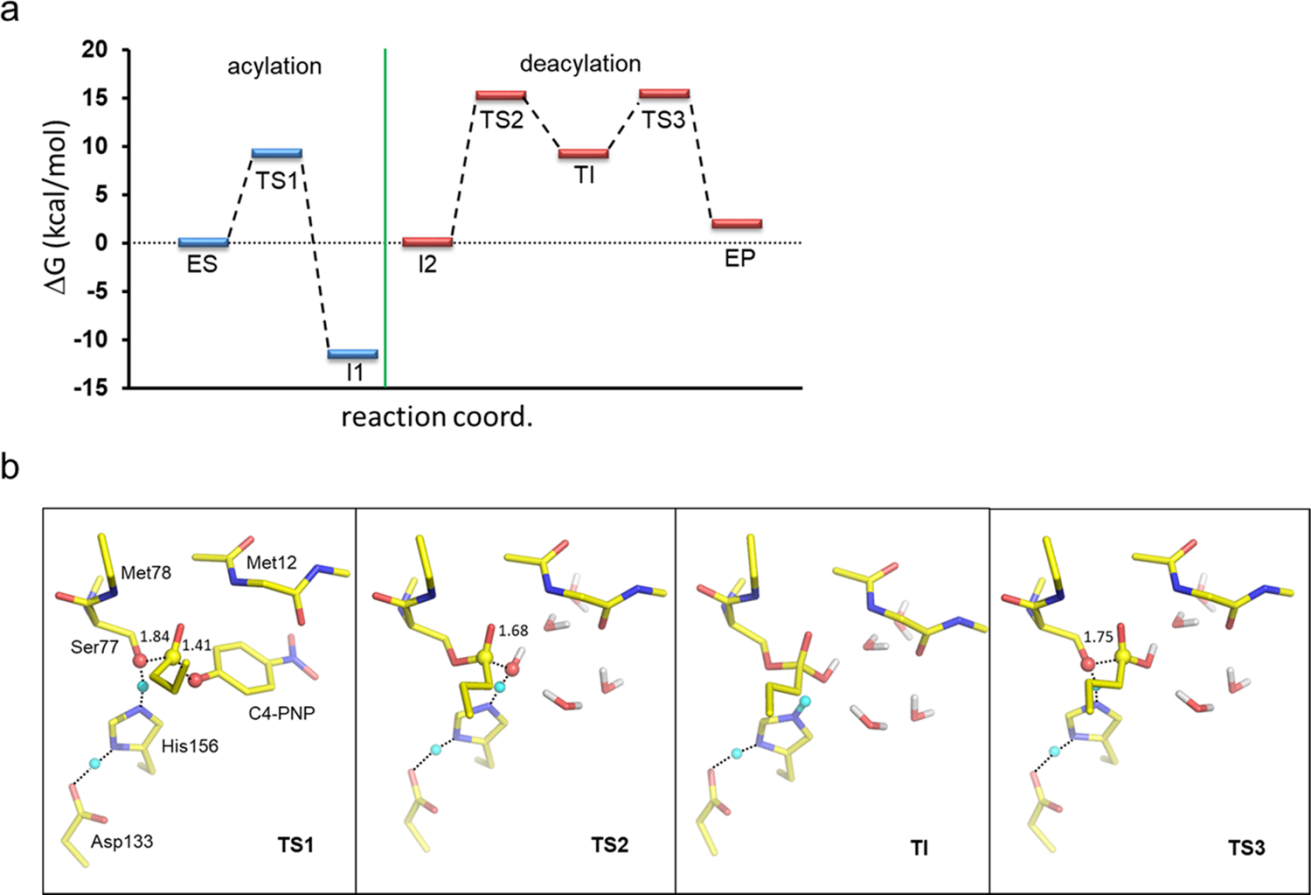
(a) Reaction free energy profile for pLipA obtained from the QM/MM simulations by exponential averaging of 20 replicas for both the acylation and deacylation pathways. The free energy level of the intermediate I2 (resulting from the dissociation of *p*-nitrophenol from I1) is arbitrarily set to zero to show the barrier height more clearly for the deacylation reaction. The arithmetic standard errors of the mean (s.e.m.) are ≤0.71 kcal/mol for the energies of the acylation step and ≤1.37 kcal/mol for the deacylation step. (b) View of the three transition states and tetrahedral intermediate along the overall reaction path, where partially formed/broken bonds are indicated with dashed lines and heavy atom distances are indicated. Water molecules interacting with the reaction center in the deacylation reaction are also shown.

The resulting free energy profiles and transition structures from the QM/MM calculations are shown in Figure 5, and the reaction paths as a function of the key distances are given in Figure S4. For the first acylation step, there is thus no stable tetrahedral intermediate and the nucleophilic attack of Ser77 on the substrate is concerted with its deprotonation by His156 and bond breaking to the leaving group but asynchronous with the latter. The transition state here is thus early, with little bond breaking to the leaving group (Figure S4a). The barrier for this step (TS1) is quite low and predicted to be about 9.3 kcal/mol, with the resulting acyl-enzyme intermediate (I1) clearly downhill from the reactant state (ES). The intermediate state I1 still has the *p*-nitrophenyl leaving group bound in the active site, and its dissociation from the enzyme is expected to be slightly uphill in free energy, depending on its binding affinity. In about 40% of the QM/MM replicas, we observe proton transfer from His156 to the phenolate leaving group, indicating that the effective p*K*_a_’s of the two groups are almost matched in the active site and that restoring the neutral histidine side chain for the subsequent deacylation comes at a low energetic cost. Note that the unperturbed p*K*_a_’s of histidine and *p*-nitrophenol in water are both about 7, and it thus seems that the enzyme does not significantly change this balance. Here, the efficient catalysis of the acylation step with the PNP-C4 substrate is primarily due to the stabilization of the developing negative charge on the ester and leaving group oxygens in TS1 by the Met12 and Met78 oxyanion hole and the protonated His157, respectively (Figure 5b). However, the fact that *p*-nitrophenyl is a good leaving group and considerably better than the Ser78 oxyanion in the subsequent deacylation step also contributes to the low barrier for acylation.

The deacylation step is thus predicted to have significantly higher free energy barriers than the preceding acylation reaction. The QM/MM calculations for the deacylation reaction use the butyrate ester of Ser77, with *p*-nitrophenol already dissociated from the active site, as the starting point. Hence, this intermediate (I2) is likely somewhat higher in energy than I1, where the difference is dictated by the binding free energy of *p*-nitrophenol. However, the dissociation of *p*-nitrophenol is not expected to carry a large free energy penalty since the leaving group is rather hydrophilic and solvent-exposed, with no obvious favorable interactions in the binding site. For the deacylation reaction, the QM/MM calculations identify a high-energy tetrahedral intermediate (TI) that is flanked by the two transition states corresponding to the nucleophilic attack by water (TS2) and bond breaking to the leaving Ser78 side chain (TS3). The predicted activation barriers measured from I2 are 15.35 and 15.52 for TS2 and TS3, respectively (Figure 5a). Hence, the breakdown of the TI is predicted to be the rate-limiting step, albeit by only 0.2 kcal/mol, which is within our error bars. In estimating the final energetics, we used exponential averaging of the individual QM/MM path energies, which has been shown to give more accurate values for barriers when a limited number of conformations are sampled.^42^ If one instead uses plain arithmetic averages, the barrier heights naturally increase by a few kcal/mol and TS3 then becomes higher than TS2 by 2.6 kcal/mol.

The QM/MM calculations thus predict that the overall hydrolysis reaction is rate-limited by the deacylation step. The corresponding calculated free energy barrier (TS3) of 15.5 kcal/mol is, in fact, in almost quantitative agreement with the barrier for *k*_cat_ of Δ*G*^⧧^ = 14.6 kcal/mol derived from experimental measurements and burst kinetics also yields deacylation as the rate-limiting step (see below). As for the acylation step, catalysis of the deacylation reaction also mainly originates from efficient stabilization of the two transition states by the oxyanion hole made up from the Met12 and Met78 backbone amides, together with protonation of the Ser77 leaving group by the positively charged His156 in TS3, i.e., breakdown of the TI (Figure 5b). It is also noteworthy that the nucleophilic water molecule, both in the attack on the acyl-enzyme (TS2) and in the breakdown of the TI, is stabilized by two hydrogen-bonded chains of solvent molecules that provide both an H-bond donor and an acceptor to the nucleophilic water (Figure 5b). The prediction of a rate-limiting deacylation step in pLipA is also consistent with experimental data for related lipases and esterases that share the same conserved catalytic triad.^43,44^ Hence, as expected, there seems to be nothing special about the pLipA reaction mechanism with regard to cold-adaptation.

### Pre-Steady-State Kinetics

**Figure 6:**
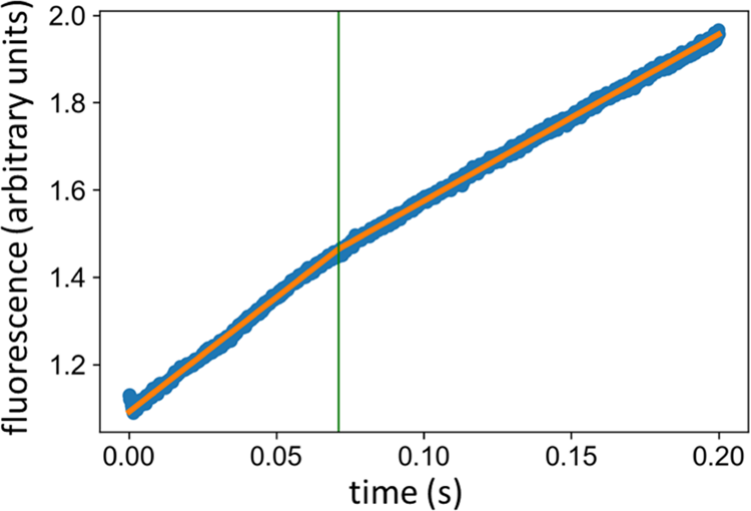
Stopped-flow experiments of pLipA with the fluorescent substrate 4-methylumbelliferyl butyrate shows a significant burst effect. A piecewise linear fit (orange line) identifies two segments, a pre-steady-state burst with higher rates and a slower steady-state, consistent with deacylation being the rate-limiting step.

Based on the observed phosphoester intermediate from the hydrolysis of paraoxon-ethyl, we expected the hydrolysis of other esters to also proceed through standard acylation and deacylation steps. To determine which of these steps is rate-limiting, pre-steady-state kinetics was employed. The fluorescent substrate 4-methylumbelliferyl butyrate forms a fluorescent product upon hydrolysis as the 4-methylumbelliferone group is released, analogous to the absorbance produced by the release of *para*-nitrophenyl from paraoxon-ethyl. The stopped-flow experiments clearly show burst kinetics consistent with deacylation being the rate-limiting step (Figure 6). Since the fluorescence is only formed when 4-methylumbelliferone is released, it is clear that no signal will be generated before the acylation has taken place, and, after that, no further fluorescence will arise before the deacylation has taken place. Thus, the burst indicates that the barrier for acylation is low and that the barrier for deacylation is higher.

**Figure 7:**
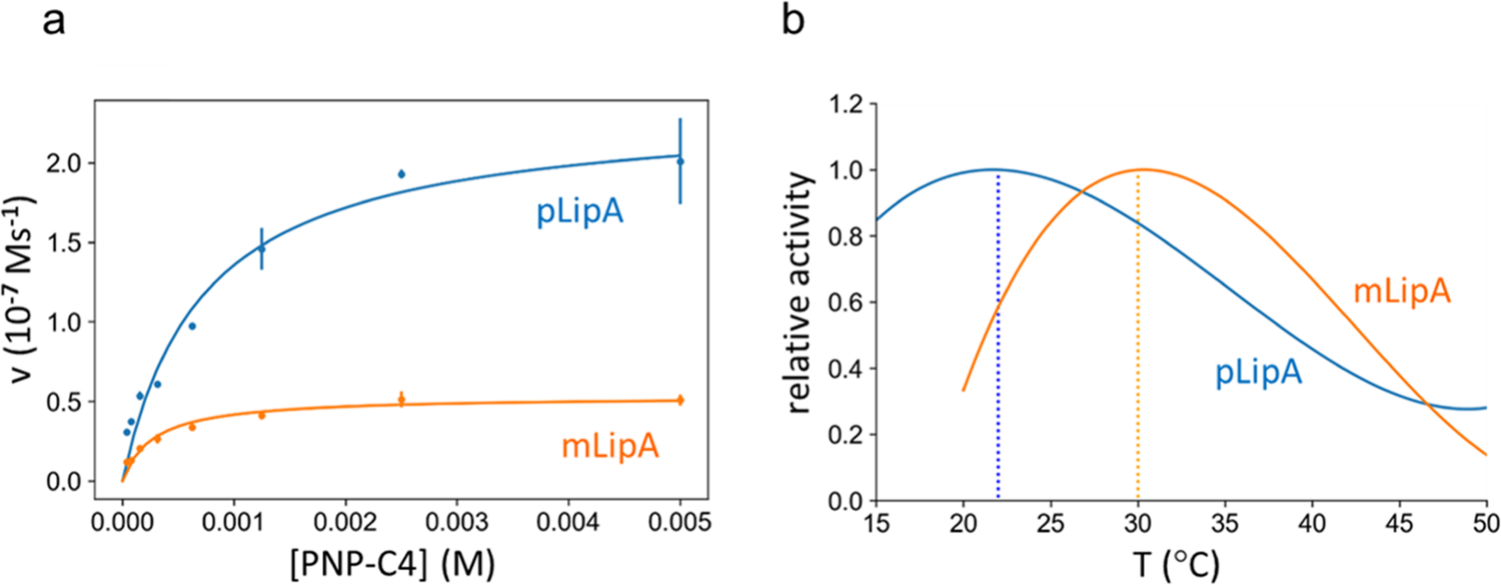
(a) Steady-state kinetics performed at 25 °C shows that pLipA (*K*_M_ = 0.7 ± 0.1 mM, *k*_cat_ = 156 ± 9 s^–1^) is more active than mLipA (*K*_M_ = 0.27 ± 0.04 mM, *k*_cat_ = 35 ± 1 s^–1^). (b) Temperature-ramping experiments with the chromogenic substrate *p*-nitrophenyl butyrate show a temperature optimum of 22 °C for pLipA and 30 °C for mLipA.

Steady-state kinetics with the chromogenic *p*-nitrophenyl butyrate substrate, which releases the yellow para-nitrophenoxide ion upon hydrolysis, was measured at 25 °C. The results show that pLipA is more active than mLipA at room temperature, with a *k*_cat_ value of 156 ± 9 s^–1^ compared to mLipA with a *k*_cat_ of 35 ± 1 s^–1^ (Figure 7a). To examine the difference in the temperature dependence of catalysis between pLipA and mLipA, a temperature ramping experiment between 15 and 50 °C, with a temperature gradient of 1 °C/min, was carried out with the *p*-nitrophenyl butyrate substrate at saturating conditions (Figure 7b). This demonstrates that pLipA is substantially more cold-adapted than mLipA and that it displays the usual characteristics of a higher activity at low temperature and a shift of its optimum toward this regime. Hence, the cold-adapted pLipA has a temperature optimum of 22 °C, while the mesophilic mLipA has an optimum of about 30 °C, using the same temperature ramping assay. These values agree fairly well with the earlier separate measurements for pLipA and mLipA.^9,10^

## Conclusions

The small lipases from *B. subtilis* and the arctic bacterium *B. pumilus* provide an interesting pair of mesophilic-psychrophilic enzymes for analyzing the structural origin of cold-adaptation in detail. Since the two enzymes are very similar in sequence, only a limited number of mutations are apparently needed to drastically change the temperature adaptation of these lipases. The crystallographic structures of pLipA determined here also show a remarkable similarity to mLipA, with the only structural variations basically located at the enzyme surface. Our QM/MM calculations clearly predict that the deacylation part of the catalytic reaction is rate-limiting with PNP-C4 as the substrate. This is confirmed by stopped-flow experiments that show a pre-steady-state burst with a higher rate than that of the slower steady-state. The steady-state kinetics further shows that pLipA has approximately a 5-fold higher value of *k*_cat_ than mLipA at room temperature, in accordance with several other examples of such differently adapted enzyme pairs.^1−4^ The adaptation of pLipA to low temperature and its distinctly different behavior compared to mLipA is also confirmed by our temperature ramping experiments. It is noteworthy that the only mutation in the substrate binding site is the I12M substitution, while other mutations are located further away on the enzyme surface. It is thus very difficult, if not impossible, to understand the different temperature dependence of the two enzymes solely based on their 3D structures, although it is likely that the combined effect of the mutations is to alter the activation enthalpy and entropy in a similar way to that observed in other cold-adapted enzymes.^2−4^ However, as noted above, with the structural and kinetic information obtained herein, it should now be possible to use more efficient computer simulation methods^4,12−15^ to explore and predict the temperature dependence of mutated and chimeric variants of these lipases, opening the way for rational design of the thermal properties.

## Supporting Information Available

Time evolution of backbone RMSD during MD simulation (Figure S1); comparison of the crystal structures of pLipA and mLipA (Figure S2); benchmark of hybrid functionals (Figure S3); and detailed reaction paths from the QM/MM calculations for the acylation and deacylation reactions (Figure S4) (PDF)

## Accession Codes

The protein structures presented herein have been deposited in the Protein Data Bank as entries 7R1K and 7R25. Input files for NAMD and ORCA calculations and PDB files for initial structures and stationary points have been deposited to the Zenodo repository as DOI:10.5281/zenodo.6382055.
