# Supplementary material for: Structure and Mechanism of a Cold-Adapted Bacterial Lipase
Source: Biochemistry. 2022 May 3;61(10):933–42. doi: 10.1021/acs.biochem.2c00087 (PMC9118546; doi:10.1021/acs.biochem.2c00087)
Supplement: Supplementary file 1 — bi2c00087_si_001.pdf [file bi2c00087_si_001.pdf]

Supporting Information

Structure and Mechanism of a Cold-Adapted Bacterial Lipase

Florian van der Ent,[1,] Bjarte A. Lund,[1,2] Linn Svalberg,[1] Miha Purg,[1] Ghislean Chukwu,[3] Mikael Widersten,[3] Geir V. Isaksen,[2] Bjørn O. Brandsdal,[2,*] Johan Åqvist[1,2,*]

[1]*Department of Cell & Molecular Biology, Uppsala University, Biomedical Center, SE-751 24 Uppsala, Sweden*

[2]*Department of Chemistry, University of Tromsø – The Arctic University of Norway, N9037 Tromsø, Norway*

[3]*Department of Chemistry – BMC, Uppsala University, Biomedical Center, SE-751 23 Uppsala, Sweden*

[*]Corresponding authors

e-mail: aqvist@xray.bmc.uu.se, bjorn-olav.brandsdal@uit.no

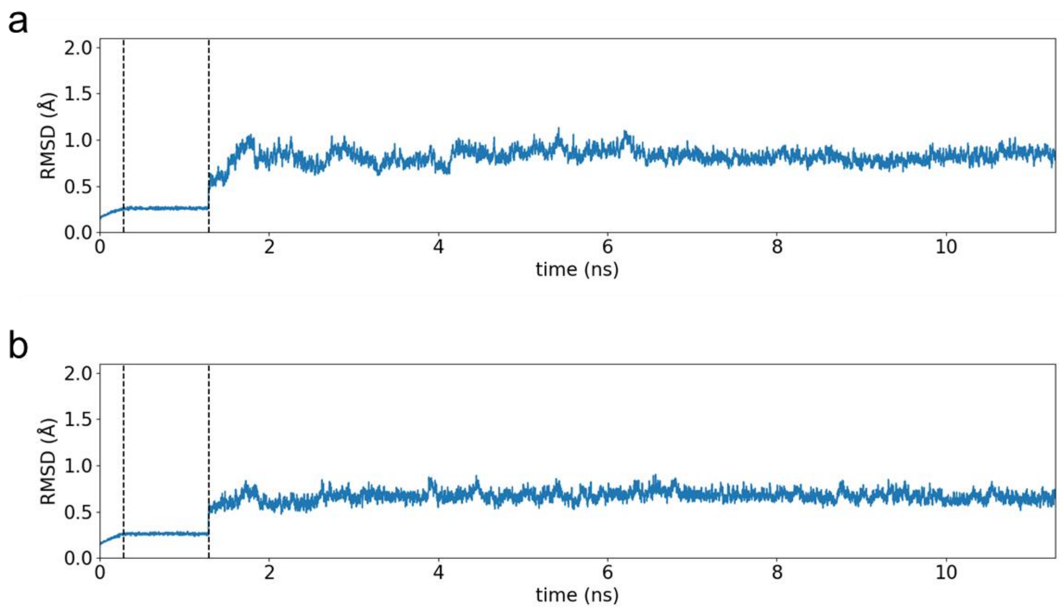

Figure S1. Time evolution of the backbone RMSD from the initial structure for one of the MD simulations of (**a**) the enzyme-substrate complex and (**b**) the acyl-enzyme. The first dashed line denotes the end of the heating phase and the second dashed line denoted the end of the equilibrium phase with backbone restraints applied.

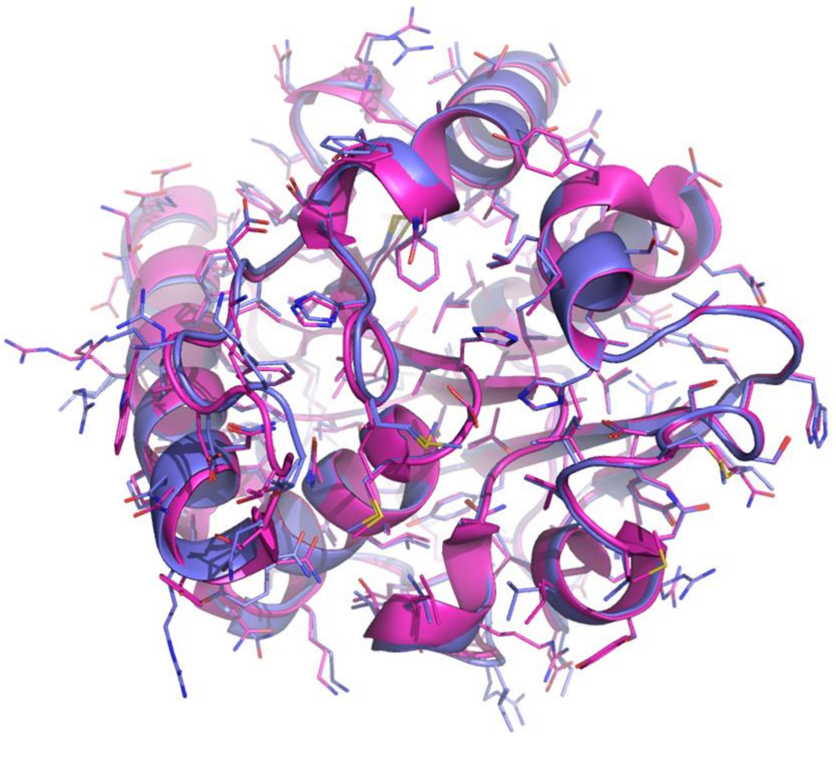

Figure S2. Comparison of the crystal structures of the apo forms of pLipA (blue) and mLipA (pink).[19]

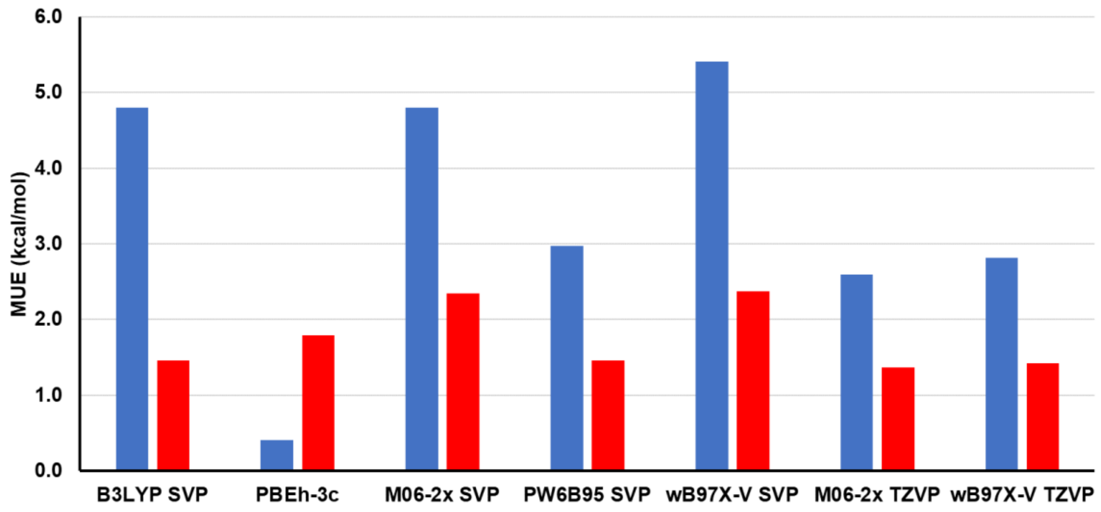

Figure S3. Benchmark of five medium and two higher cost (def2-TZVP) hybrid functionals with respect to the RI-DSD-PBEB95-D3BJ double hybrid functional with the def2-QZVPP basis set. The mean unsigned error (MUE) is calculated for barrier heights (blue bars) and relative reaction energies (red bars) from the first two replicas of both the acylation and deacylation reactions.

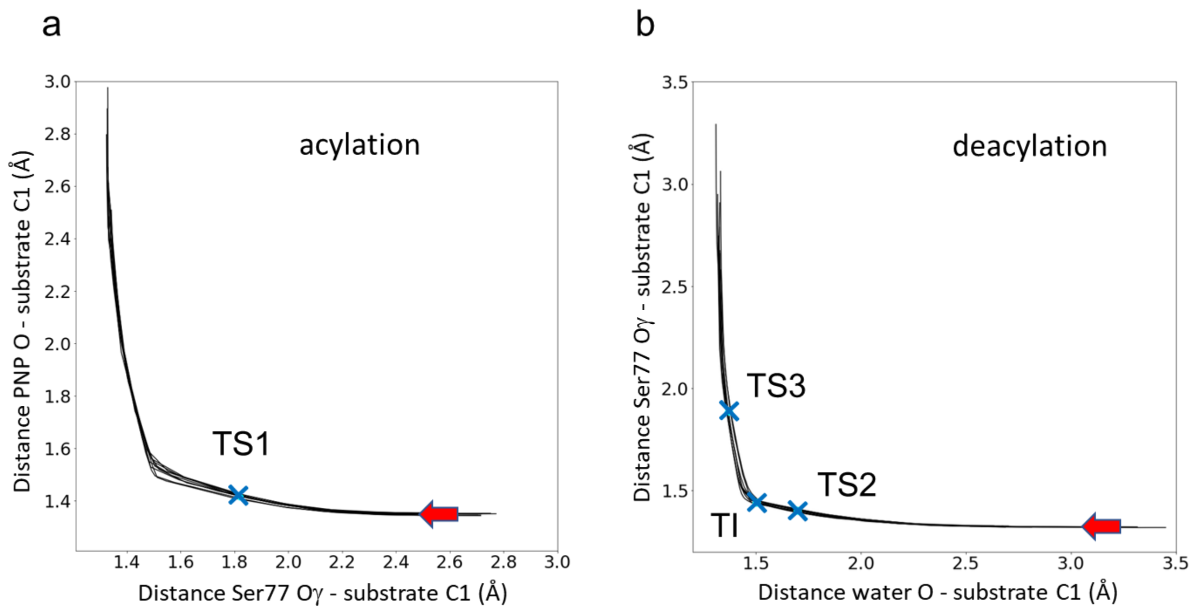

Figure S4. View of the QM/MM reaction paths for the (**a**) acylation and (**b**) deacylation reactions in pLipA (the 20 replicas are overlayed in each case). The geometrical coordinates correspond to the bond formed between the heavy atoms (*x*-axis) and the bond broken to the leaving group (*y*-axis). Stationary points are indicated.
